# PRÉVALENCE ET FACTEURS ASSOCIÉS À LA GALE EN MILIEU CARCÉRAL À DOSSO, NIGER

**DOI:** 10.48327/mtsi.v3i3.2023.398

**Published:** 2023-07-06

**Authors:** Moussa HAROUNA, Abdoul Kadir IBRAHIM MAMADOU, Mazou HAMADOU, Saraye OUSMANE, Kadidia ISSA ABDOU, Inouss ALI, Oumalkhair ZAKARI SIDI

**Affiliations:** 1 Service de dermatologie-vénéréologie, Centre hospitalier régional de Dosso, Niger; 2 Service de médecine interne, Centre hospitalier régional de Dosso, Niger; 3 Service de pédiatrie, Centre hospitalier régional de Maradi, Niger; 4 Service de dermatologie-vénéréologie, Hôpital national de Niamey, Niger; 5 Service de dermatologie-vénéréologie, Hôpital national de Zinder, Niger; 6 Service de dermatologie-vénéréologie, Hôpital national Amirou Boubacar Diallo de Niamey, Niger

**Keywords:** Gale, *Sarcoptes scabiei*, Dermatose prurigineuse, Prison, Dosso, Niger, Afrique subsaharienne, Scabies, *Sarcoptes scabiei*, Pruritic dermatosis, Prison, Dosso, Niger, Sub-Saharan Africa

## Abstract

**Introduction:**

La gale ou scabiose est une dermatose contagieuse, prurigineuse, cosmopolite, très répandue notamment dans les collectivités humaines, due au parasitisme par un acarien *Sarcoptes scabiei* var *hominis.* Un défaut d'hygiène et le surpeuplement à l'intérieur des habitations sont des facteurs favorisants. Le but de notre étude était de dégager la prévalence et les facteurs associés à la survenue de la gale humaine en milieu carcéral.

**Matériel et méthodes:**

Il s'agit d'une étude prospective transversale descriptive menée en novembre 2022 à la prison civile de Dosso (Niger). Le diagnostic était clinique.

**Résultats:**

Sur un total de 352 détenus, 43 (38 hommes et 5 femmes) avaient la gale soit une prévalence de 12%. L'âge moyen était de 33 ans avec des extrêmes de 18 et 62 ans. La plupart des détenus, 86%, étaient dans des cellules de plus de 20 détenus, et parmi ceux atteints de gale, 42% se lavaient 2 fois dans la journée, 81% changeaient ensuite d'habits et 74% utilisaient régulièrement le savon pour la toilette. Les sièges des lésions étaient principalement les organes génitaux externes (37%), les fesses (21%) et la face interne des cuisses (16%).

**Conclusion:**

La gale humaine existe en milieu carcéral à Dosso. Les conditions d'hygiène et de détention étaient significativement associées à sa survenue. La sensibilisation sur l'hygiène et un suivi dermatologique régulier des détenus est nécessaire. Les données de notre étude peuvent contribuer à l'amélioration des soins en milieu carcéral. Les autorités pénitentiaires de la région devraient faire de l'amélioration des conditions d'hygiène et de détention une priorité.

## Abstract (en)

**Background:**

Scabies is a contagious, pruritic, cosmopolitan dermatosis, widespread especially in human communities, due to parasitism by a mite *Sarcoptes scabiei* var *hominis* A lack of hygiene and overcrowding inside the houses are favourable factors. The aim of our study was to determine the prevalence and factors associated with the occurrence of human scabies in the prison environment in Dosso (Niger).

**Materials and methods:**

Prospective descriptive cross-sectional study conducted in November 2022 in the civil prison of Dosso on authorization of the prison administration. The collected data were sociodemographic characteristics, personal history of pruritus and its characteristics, risk factors related to hygiene and conditions of incarceration, examination performed by a dermatologist to look for clinical signs of scabies. The diagnosis was retained in any inmate presenting one or more elements at the interrogation and at the physical examination.

**Results:**

Out of a total of 352 inmates, 43 (38 males and 5 females) had scabies, a prevalence of 12%. The average age was 33 with extremes of 18 and 62 years. Of the scabies patients, 9% reported a history of intense, sleepless pruritus prior to incarceration. Most of the inmates, 86%, were in cells with more than 20 inmates. Among those with scabies, 42% showered twice a day, 81% changed clothes after showering and 74% used soap regularly for washing. The topography of the lesions was external genitalia (37%), buttocks (21%), inner thighs (16%), interdigital spaces (9%), elbows (9%) and wrists (7%). The inmates were informed about the disease, treatment information was explained to them, and they attended a session on hygiene promotion. All cases of scabies detected were prescribed a local treatment based on benzyl benzoate lotion 25%.

**Discussion/conclusion:**

Human scabies is common in the prison environment in Dosso. Too often, scabies goes undetected and is confused with other pruritic dermatoses, such as eczema or atopic dermatitis. Prison medical staff should be trained in scabies in order to prevent scabies outbreaks. The authorities in the region should make the improvement of hygiene and detention conditions in prisons their priority.

## Introduction

La gale ou scabiose est une dermatose contagieuse, prurigineuse, cosmopolite, très répandue notamment dans les collectivités humaines, due au parasitisme par un acarien *Sarcoptes scabiei* var *hominis.* Un défaut d'hygiène, la pauvreté et le surpeuplement à l'intérieur des habitations sont des facteurs favorisants. La gale est considérée par l'OMS comme une Maladie tropicale négligée (MTN) depuis 2017 [10]. Un prurit sévère, surtout la nuit, est le symptôme le plus précoce et le plus courant. La prévalence mondiale a été estimée à environ 300 millions de cas par an [3, 9]. Les prisons sont un réservoir potentiel pour diverses maladies de la peau, en particulier les infections [5]. Beaucoup de travaux sur la gale en milieu carcéral ont été réalisés et la prévalence varie selon le pays : 32% au Cameroun, 13% au Burkina Faso, 66% au Sénégal et 2% en Pologne [1,2,4,11]. L'absence de données, dans notre contexte, nous a conduits à mener cette étude. Le but était de déterminer la prévalence et les facteurs associés à la survenue de la gale humaine en milieu carcéral à Dosso (Niger).

## MATÉRIELS ET MÉTHODE

Nous avons conduit une étude prospective transversale descriptive au mois de novembre 2022 à la prison civile de Dosso sur autorisa tion de l'administration pénitentiaire. Dosso est le chef-lieu de région situé à 140 km à l'est de la capitale Niamey. Tous les détenus ont été informés au préalable. Leur consentement éclairé a été obtenu oralement. L'intimité et le secret médical ont été préservés dans la mesure du possible dans un milieu carcéral. Au total il y avait 352 détenus. Nous avons procédé à un interrogatoire afin de recueillir les caractéristiques sociodémographiques, les antécédents personnels de prurit et ses caractéristiques, les facteurs de risques relatifs à l'hygiène et l'assainissement (fréquence des toilettes, utilisation de savon, changement de vêtements après la toilette) ainsi que les conditions d'incarcération (nombre de détenus par cellule et durée d'incarcération). Un examen physique a été réalisé dans l'infirmerie de la prison par un dermatologue assisté d'un médecin généraliste et d'un technicien supérieur en dermatologie afin de rechercher des lésions spécifiques (vésicules perlées, nodules scabieux, sillons scabieux, topographie des lésions) et non spécifiques (stries de grattage, excoriations, papules, chancres). Le diagnostic de la gale a été retenu chez tout détenu présentant un ou plusieurs éléments de l'interrogatoire et de l'examen clinique. Les données, recueillies sur une fiche individuelle anonyme, ont été saisies avec le logiciel SPSS. L'analyse des données a été faite par le même logiciel et le seuil de significativité des variables qualitatives et quantitatives étudiées a été considéré pour une valeur de p < 0,05.

## Résultats

Sur un total de 352 détenus (341 hommes et 11 femmes), 43 souffraient de la gale (38 hommes et 5 femmes) soit une fréquence de 12% (Tableau I). L'âge moyen était de 33 ans avec des extrêmes de 18 et 62 ans. Tous les détenus déclaraient dormir sur une natte et la durée d'incarcération était de 6 mois à un an pour 61% des détenus (Tableau II).

**Tableau I:** Répartition de l'effectif total de prisonniers par tranche d'âge et par sexe Breakdown of total number of prisoners by age group and gender

Âge	Sexe		Total
	**M**	**F**	
**Moins de 20 ans**	28	2	30
**20 - 29 ans**	146	4	150
**30 - 39 ans**	87	3	90
**40 - 49 ans**	44	2	46
**50 - 59 ans**	25	0	25
**60 ans et plus**	11	0	11
**Total**	341	11	352

**Tableau II:** Caractéristiques sociodémographiques des 43 patients atteints de gale humaine Sociodemographic characteristics of the 43 patients with human scabies

Âge	Effectif
moins de 20 ans	6
20 - 29 ans	16
30 - 39 ans	9
40 - 49 ans	5
50 - 59 ans	6
60 ans et plus	1
**Sexe**	
M	38
F	5
**Durée d'incarcération**	
moins de 6 mois	9
6 mois à 1 an	26
plus d'1 an	8

Parmi les 43 patients atteints de gale, 9% déclaraient avoir un antécédent de prurit intense insomniant avant l'incarcération, 42% se lavaient avec de l'eau dans un seau deux fois dans la journée, 74% utilisaient régulièrement le savon pour leur toilette, 81% changeaient ensuite de vêtements. La majorité des détenus, 86%, étaient dans des cellules de plus de 20 détenus la nuit. Les lésions spécifiques de la gale ont été trouvées chez 29 détenus (67%) et les lésions non spécifiques chez 14 détenus (33%). La topographie des lésions était : organes génitaux externes (37%), fesses (21%), face interne des cuisses (16%), espaces interdigitaux (9%), coudes (9%) et poignets (7%) (Tableau III). La durée d'incarcération est apparue liée à la fréquence de la maladie de manière négative (moins de cas chez les détenus de longue durée) (Tableau IV).

**Tableau III:** Répartition des 43 cas de gale en fonction du type et du siège des lésions Breakdown of total number of prisoners by age group and gender

	Effectif	Pourcentage%
**Lésions spécifiques**	
vésicules perlées	12	28
nodules scabieux	11	25
Sillons scabieux	6	14
**Lésions non spécifiques**		
stries de grattage	5	12
papules	4	9
papules excoriées	5	12
**Siège des lésions**		
organes génitaux externes	16	37
face interne des cuisses	7	16
fesses	9	21
espaces interdigitaux	4	9
coudes	4	9
poignets	3	7

**Tableau IV:** Facteurs associés à la survenue de la gale chez les détenus à Dosso Factors associated with the occurrence of scabies among inmates in Dosso

	**Gale**		**Khi^2^ de Pearson**	**P-value**
	Oui	Non		
**Fréquence de toilette**				
1 fois par jour	1	8	25,864	< 0,05
2 fois	18	213		
3 fois	18	82		
4 fois	5	6		
plus de 4 fois	1	0		
**Utilisation du savon pendant la toilette**				
oui	15	221	21,932	< 0,05
non	28	88		
**Changement de vêtements après la toilette**				
oui	33	221	0,53	NS
non	10	88		
**Nombre de détenus dans la cellule**				
moins de 20	5	36	0,00	NS
plus de 20	38	273		
**Durée d'incarcération**				
moins de 6 mois	9	24	27,412	<0,05
6 mois à 1 an	26	99		
plus d'1 an	8	186		

Tous les détenus ont été informés sur la maladie. Les informations relatives au traitement leur ont été expliquées et ils ont suivi une session sur la promotion de l'hygiène. Tous les cas de gale ont bénéficié de la prescription d'un traitement local à base de benzoate de benzyle en lotion à 25%. Des ordonnances ont été remises à l'administration pénitentiaire car les détenus n'avaient pas de source de revenu en dehors de l'aide matérielle et financière de leur entourage. L'utilisation de ce produit a été expliquée aux détenus et au personnel de l'infirmerie.

## Discussion

Cette étude sur la gale humaine en milieu carcéral est la première dans notre contexte. Elle nous a permis de dégager d'une part une prévalence de 12% de la gale humaine en milieu carcéral et d'autre part les conditions d'hygiène et de détention comme facteurs de risque associés à la survenue de cette pathologie. C'est dans cette optique que les autorités pénitentiaires de Dosso avaient émis le besoin d'assistance d'une équipe spécialisée pour investiguer une affection dermatologique prurigineuse constatée chez des détenus. L'entretien avec les détenus et leur examen ont été facilités par l'équipe de l'infirmerie et l'usage de leur local dans la prison. Nous n'avons pas pu faire de prélèvements pour l'examen parasitologique. Cela pourrait constituer une limite de notre étude, bien que le diagnostic paraclinique de la gale humaine ne soit pas nécessaire. Dosso étant l'une des huit régions que compte le pays, ces données ne peuvent pas être extrapolées à l'ensemble du pays.

La prévalence de la gale humaine dans notre étude était inférieure à celles trouvées dans des prisons au Sénégal (66%), au Cameroun (32%), au Pakistan (43%) ou en Iran (55%) [6]. La relativement faible prévalence observée peut s'expliquer par le fait que certains cas de gale avaient été diagnostiqués et pris en charge à l'hôpital avant l'étude.

Nous avons noté des relations statistiquement significatives entre les conditions d'hygiène telles que la fréquence des toilettes (p < 0,05), l'utilisation du savon (p < 0,05), la durée d'incarcération (p < 0,05) et la survenue de la gale humaine. Ces relations sont surprenantes et nullement explicatives. Les cas de gale semblent se laver plus fréquemment, peut-être à cause du prurit, et être plus fréquents parmi les prisonniers avec un séjour court. D'autres facteurs de risques liés aux conditions d'hygiène et de détention ont été rapportés dans plusieurs populations carcérales, à savoir le nombre de personnes par lit, le nombre de détenus par cellule, et le partage d'habits [3,4,7,8]. Les conditions d'hygiène et de détention dans les prisons en Afrique subsaharienne sont souvent mauvaises, exposant la population carcérale à diverses maladies infectieuses dont la gale [12]. Les données de notre étude peuvent contribuer à améliorer les soins en milieu carcéral. Les autorités pénitentiaires de la région devraient faire de l'amélioration des conditions d'hygiène et de détention dans les prisons leur priorité. L'éducation des prisonniers, à travers une sensibilisation accrue, un diagnostic précis lors de consultations dermatologiques régulières et un traitement rapide sont des outils pour lutter contre la gale et prévenir sa propagation dans les établissements pénitentiaires.

À l'issue de cette étude, l'administration pénitentiaire a reçu un don de perméthrine crème en lieu et place du benzoate de benzyle prescrit initialement. Tous les cas de gale ont été traités le même jour et leurs habits et nattes lavés dans l'eau à plus de 60 °C. Le traitement à base d'ivermectine a été envisagé, mais non encore réalisé du fait non seulement du manque de moyens financiers des détenus mais aussi de la non-disponibilité du produit.

## Conclusion

La prévalence de la gale dans la prison de Dosso était de 12%, avec une prédominance chez les jeunes adultes de 20 à 29 ans. La gale est un problème de santé courant en milieu carcéral. Les autorités pénitentiaire et judiciaire doivent faciliter des séances de dépistage et de prise en charge de la gale dans ce milieu.

## Liens D'intérêts

Les auteurs ne déclarent aucun conflit d'intérêts.

## Contribution Des Auteurs

M. Harouna et O. Zakari Sidi ont dirigé l'étude, rédigé le protocole de recherche, collecté et analysé les données et rédigé le manuscrit. A. K. Ibrahim Mamadou a fourni la bibliographie et effectué une lecture critique. M. Hamadou, S. Ousmane, K. Issa Abdou et I. Ali ont procédé à une lecture critique et suggéré des corrections finales. Tous les auteurs ont lu et approuvé le manuscrit final.

## Remerciements

Les remerciements s'adressent particulièrement au directeur du Centre hospitalier régional de Dosso, aux autorités judiciaires de Dosso ainsi qu'au directeur de l'administration pénitentiaire de Dosso et à son équipe.
